# Adequate Antithrombin III Level Predicts Survival in Severe COVID-19 Pneumonia

**DOI:** 10.7759/cureus.18538

**Published:** 2021-10-06

**Authors:** Deepti Joshi, Sarat Manohar, Garima Goel, Saurabh Saigal, Abhijit P Pakhare, Abhishek Goyal

**Affiliations:** 1 Pathology and Laboratory Medicine, All India Institute of Medical Sciences, Bhopal, Bhopal, IND; 2 Anaesthesiology, All India Institute of Medical Sciences, Bhopal, Bhopal, IND; 3 Community and Family Medicine, All India Institute of Medical Sciences, Bhopal, Bhopal, IND; 4 Pulmonary Medicine and Tuberculosis, All India Institute of Medical Sciences, Bhopal, Bhopal, IND

**Keywords:** anticoagulant, endogenous, heparin, covid-19, antithrombin

## Abstract

Critically ill patients with COVID-19 are at an increased thrombotic risk, hence thromboprophylaxis with heparin is considered mandatory. Antithrombin III (ATIII) is the most potent endogenous anticoagulant and is required for the clinical efficacy of heparin. Profound hypercoagulable and inflammatory state associated with COVID-19 can result in decreased ATIII levels and ineffective heparin treatment resulting in increased mortality. The present study evaluated ATIII levels in critically ill patients of COVID-19 and correlated them with other coagulation parameters and disease outcomes. A retrospective review of those critically ill COVID-19 patients was performed who were on a therapeutic dose of low molecular weight heparin (LMWH) and had serial measurements of ATIII, anti-factor Xa (antiFXa) assay and other routine coagulation parameters. A total of 27 critically ill COVID-19 patients were identified, out of these, 12 survived and 15 had disease-induced mortality. ATIII levels were found to be significantly lower in non-survivors on the third day of serial measurement along with worsening of other coagulation parameters. AntiFXa levels were found to be higher in non-survivors as compared to survivors. Further studies are required to establish ATIII as a prognostic marker and to determine the utility of monitoring antiFXa levels in COVID-19 patients on LMWH therapy.

## Introduction

Coronavirus disease 2019 (COVID-19) has emerged as a major global health crisis and has so far affected millions of individuals all across the globe and resulted in more than 3.5 million deaths [1,2]. The disease is caused by severe acute respiratory syndrome coronavirus 2 (SARS-CoV-2) which belongs to the beta coronavirus family and is highly infectious [3]. The manifestations of the disease vary from a mild respiratory tract infection to a systemic severe form of the disease characterised by acute lung injury, acute respiratory distress syndrome (ARDS), shock, multiple organ dysfunction and disseminated intravascular coagulation (DIC) [4]. Excessive inflammatory response, endothelial dysfunction and thrombosis have emerged as dominant pathophysiological features that determine the course of this disease [3].

Coagulopathy in individuals with severe COVID-19 has important differences as compared to that seen in severe inflammatory response syndrome (SIRS) and sepsis. COVID-19 coagulopathy is characterised by high levels of D-dimer, but unlike sepsis-associated coagulopathy, it shows only minimal prolongation of prothrombin time (PT), activated partial thromboplastin time (APTT) and mild thrombocytopenia. Moreover, thrombosis is a dominant phenomenon in COVID-19 infection rather than bleeding [5]. This phenomenon has led most guidelines to recommend the use of anti-coagulants, either unfractionated heparin (UH) or low molecular weight heparin (LMWH), for all moderate to severely ill patients with COVID-19. Despite heparin prophylaxis, a substantial number of these patients develop thromboembolic complications [6]. Heparin mediates its effect through antithrombin III (ATIII), which is one of the most powerful natural anticoagulants. Upon binding to heparin, its propensity to inhibit thrombin and factor Xa is increased manifold. Inflammation can significantly reduce the levels of ATIII resulting in decreased efficacy of heparin. Both reduced ATIII levels and the phenomenon of heparin resistance in COVID-19 patients have been reported in few studies [7,8]. However, the exact relationship between ATIII titres and other markers of disease progression is not yet established [7].

To explore these concerns, we evaluated ATIII levels in critically ill COVID-19 patients and correlated these with other coagulation parameters and disease outcomes. Our research question was to determine whether decreased ATIII level is associated with mortality amongst critically ill COVID-19 patients on a therapeutic dose of LMWH.

## Materials and methods

We conducted a retrospective chart review of critically ill adult patients admitted with COVID-19 related ARDS to the Intensive Care Unit (ICU) at All India Institute of Medical Sciences, Bhopal, a tertiary care hospital in Central India. All such patients were treated with oxygen and ventilator support, high-dose corticosteroids, anti-coagulants, anti-platelets, remdesivir, antibiotics and other supportive therapies as per therapeutic protocol. Intensivists usually ordered coagulation studies on multiple occasions in many patients. We restricted the study to only those patients who had serial measurements of ATIII assay, anti-factor Xa (antiFXa) assay, PT/international normalised ratio (INR), APTT, platelets and fibrin degradation products (FDP) done on the first three days of ICU admission. This was done to eliminate information bias pertaining to laboratory measures. Information about their demography, clinical features, laboratory investigations and in-hospital survival was collected from ICU charts. The study design was approved by the Institutional Human Ethics Committee - Post Graduate Research (IHEC-PGR), AIIMS Bhopal with approval letter number 2020/PG/July/33 dated 24th February 2021 for research on human subjects, and a waiver of consent was obtained.

Laboratory procedures

Sample Collection

Samples for routine coagulation studies were received in 3.2% sodium citrate vial, with a blood-to-anticoagulant ratio of 9:1, centrifuged at 3000 rpm for 20 minutes to yield platelet-poor plasma from which subsequent tests were performed. Complete blood count (CBC) samples were received in EDTA (ethylenediamine tetraacetic acid) vials;

Coagulation Tests

All the coagulation parameters were analyzed in the Pathology department. PT and APTT were performed in the semiautomated Stago coagulometer (STart Max, Asnières sur Seine, France). Fibrinogen, FDP, antiFXa assay and antithrombin III assay were performed on Stago automated coagulometer (STA Satellite MAX, Asnières sur Seine, France). All the tests were performed using propriety reagents (Diagnostica Stago, Asnières sur Seine, France). The samples were processed within two hours of reception. Samples for antiFXa assay were received within 3-4 hours of administration of the dose of LMWH. Appropriate controls were used with all the tests. The samples for CBC were run on Mindray BC- 6800 hematology analyzer (Shenzhen, China) using propriety reagents.

Statistical analysis

Statistical analyses were conducted using the R Statistical language (version 4.0.3; R Core Team, Vienna, Austria) on Mac OS Catalina 10.15.6, using the packages ggpubr (version 0.4.0), gtsummary (version 1.4.1), ggplot2 (version 3.3.2) and tidyverse (version 1.3.0). We performed descriptive statistical analysis for all categorical and quantitative variables. The results were given as the median (interquartile range), or number (percentage), wherever appropriate. The difference between continuous and categorical variables was compared using Wilcoxon rank-sum test and Fisher exact tests/Pearson Chi-square test respectively and p<0.05 was used to classify the difference between the two groups as significant.

## Results

We identified a total of 58 patients with ARDS who were admitted for more than three days to ICU between October and November 2020. Twenty-seven out of 58 patients had an entire panel of coagulation tests done in a serial manner on three consecutive days. The median symptom-hospitalization interval of these patients was 7.5 days (IQR 6 to 9), and all patients had oxygen saturation (SpO2)/fraction of inspired oxygen (FiO2) of less than 150 on ICU admission. All patients were followed up till death or discharge from the hospital, and a total of 12 (44.4%) of these patients survived, the remaining 15 (55.6%) died. Survivors and non-survivors had similar age, gender, symptom duration, and distribution of co-morbidities. Non-survivors had worse Sequential Organ Failure Assessment (SOFA) scores on ICU admission and were more likely to require invasive mechanical ventilation during ICU stay (Table 1).

**Table 1:** Differences in clinical characteristics amongst survivors and non-survivors (n=27) ^1^ Values for these characteristics are in medians (IQR); the remaining represent n (%); ^2 ^p-value is for Wilcoxon rank-sum test for difference in medians and Chi-square test for difference in proportions

Characteristic	Survivors N = 12	Non-Survivors N = 15^1^	p-value^2^
Age^1^	54 (45, 59)	60 (54, 72)	0.092
Gender Female	4 (33%)	3 (20%)	0.665
Male	8 (69%)	12 (80%)	
Symptom admission interval (days)^ 1^	7.00 (6.00, 8.00)	8.00 (7.50, 9.00)	0.024
Number of co-morbidities			
None	5 (42%)	3 (20%)	0.324
One	3 (25%)	5 (33%)	
Two	1 (8.3%)	5 (33%)	
Three or more	3 (25%)	2 (13%)	
SOFA score on ICU admission^1^	3.00 (2.75, 4.25)	7.00 (4.50, 10.00)	0.002
Need for Invasive Ventilation			
0	11 (92%)	4 (27%)	<0.001
1	1 (8.3%)	11 (73%)	

**Figure 1:**
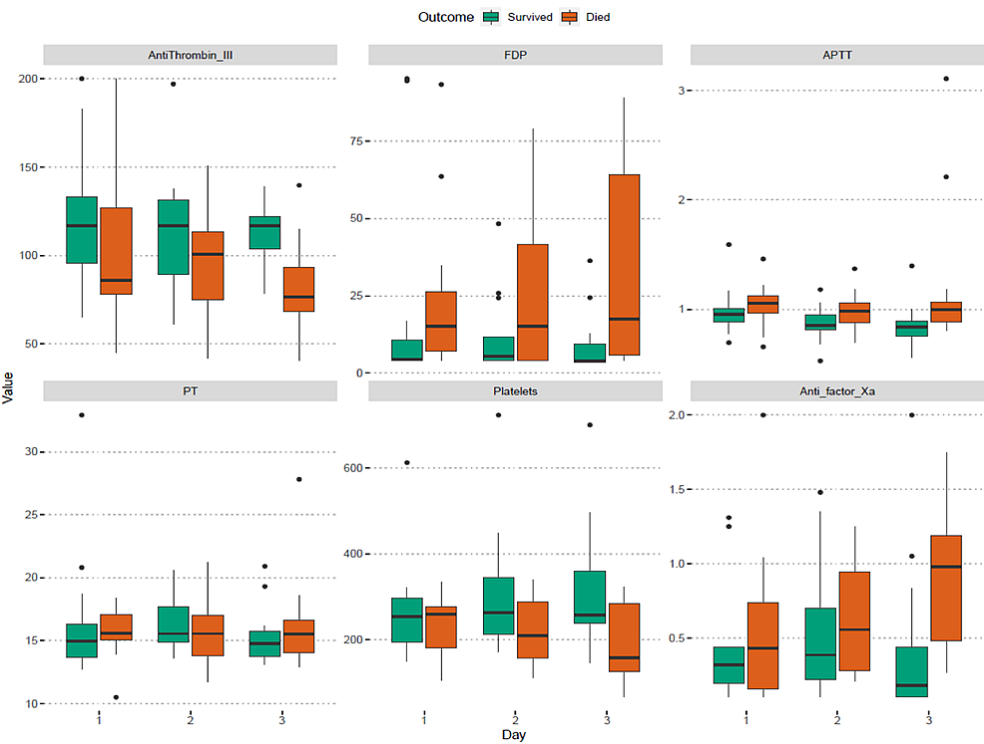
Box plot depicting differences in coagulation abnormalities in survivors and non-survivors.

ATIII levels were lower in non-survivors as compared to that in survivors on all three days of measurement and this was significant (p-value 0.002) on the third day of ICU admission. Fibrinogen level was significantly higher (p-value 0.015) in non-survivors on the day of ICU admission. All other coagulation-related laboratory parameters (platelet count, PT, INR, APTT, FDP, antiFXa) were similar in survivors and non-survivors on the first two days of ICU admission. However, on day 3, non-survivors had significantly reduced platelet counts (p-value 0.022), high FDP levels (p-value 0.014), elevated APTT (p-value 0.006) and increased antiFXa levels (p-value 0.006). A worsening trend was observed in the coagulation parameters from the first to the third day in non-survivors (Table 2, Figure 1).

**Table 2:** Differences in coagulation parameters in survivors and non-survivors (n=27) ^1^ All values are median (IQR) or n (%); ^2^ p-value is for Wilcoxon rank-sum test for medians PT: prothrombin time; INR: international normalized ratio; APTT: activated partial thromboplastin time; FDP: fibrin degradation products

Parameter^1^	Day	Survivors N = 12	Non-Survivors N = 15	p-value^2^
Platelet count (thousand/cumm)	1	254 (194, 296)	259 (186, 273)	0.542
	2	263 (212, 345)	204 (156, 283)	0.051
	3	258 (237, 359)	150 (126, 280)	0.022
PT (seconds)	1	14.95 (13.70, 16.30)	15.60 (15.03, 17.05)	0.520
	2	15.55 (14.88, 17.68)	15.55 (13.82, 17.00)	0.471
	3	14.75 (13.73, 15.75)	15.50 (14.03, 16.60)	0.554
INR	1	1.14 (1.04, 1.29)	1.18 (1.12, 1.29)	0.678
	2	1.14 (1.11, 1.35)	1.14 (0.98, 1.29)	0.341
	3	1.12 (1.05, 1.21)	1.16 (1.00, 1.21)	0.864
APTT ratio	1	0.96 (0.89, 1.01)	1.04 (0.92, 1.11)	0.283
	2	0.85 (0.81, 0.95)	1.00 (0.88, 1.05)	0.043
	3	0.84 (0.75, 0.89)	1.01 (0.91, 1.11)	0.006
FDP (microgram/ml)	1	5 (4, 11)	15 (8, 24)	0.117
	2	6 (4, 12)	15 (4, 40)	0.169
	3	4 (4, 9)	17 (7, 53)	0.014
Anti-factor Xa (IU/ml)	1	0.32 (0.20, 0.44)	0.47 (0.19, 0.77)	0.434
	2	0.38 (0.22, 0.70)	0.52 (0.30, 0.94)	0.526
	3	0.18 (0.10, 0.44)	0.96 (0.41, 1.17)	0.006
Antithrombin (%)	1	117 (95, 133)	85 (79, 121)	0.204
	2	117 (89, 131)	100 (73, 111)	0.150
	3	117 (104, 122)	76 (69, 92)	0.002
Fibrinogen (mg/dl)	1	464 (419, 578)	636 (589, 682)	0.015

## Discussion

An important finding of this study was the progressive decline in ATIII levels in non-survivors along with worsening of other coagulation parameters like APTT, FDP and platelet counts. The worsening of coagulation parameters and fall in ATIII levels may suggest an evolution of consumption coagulopathy. Consumption coagulopathy of varying degrees can complicate the course of critically ill COVID-19 patients [9,10]. ATIII is the most important endogenous inhibitor of thrombin and besides affecting intrinsic, extrinsic and common pathways of the coagulation cascade, it is also a potent anti-inflammatory agent. ATIII levels get depleted in coagulopathy due to rapid consumption after the formation of thrombin-antithrombin complexes and also due to reduced synthesis and increased degradation by neutrophil elastases in severe inflammatory conditions. In addition, the synthesis of glycosaminoglycans of the endothelial surface is reduced by inflammatory mediators thereby decreasing the efficacy of ATIII [7,11]. Reduced concentration of this factor in sepsis has been implicated in the pathogenesis of DIC and multi-organ dysfunction [12]. Heparin therapy can also result in decreased levels of ATIII [13].

ATIII deficiency has been observed in COVID-19 patients in previous studies [7,9,10,14,15]. Gazzaruso et al observed that ATIII levels were lower in non-survivors amongst hospitalized COVID-19 patients and the levels were much lower in obese individuals. However, the use of AT concentrate along with heparin was found to be associated with increased mortality [7]. Von Meijenfedt et al, Tang et al and Anakli et al reported significantly lower ATIII levels in non-survivors as compared to survivors [9,10,14]. In a recent study, Gardner et al reported ATIII deficiency in 5/19 (26%) cases and suggested the use of alternate anti-thrombotic therapies [15]. It has also been postulated that AT supplementation with fresh frozen plasma may augment heparin reactivity and improve the outcome in such patients [14]. However, most guidelines recommend against the use of AT concentrates for the treatment of sepsis-induced DIC [12].

Besides exhibiting features of COVID-19-associated coagulopathy, non-survivors were also found to have significantly increased fibrinogen levels at baseline, reflecting the response of this acute phase reactant against the COVID-19-associated cytokine storm and also indicating the profound hypercoagulable state associated with COVID-19 [4,6]. Increased levels of FDP indicate activation of the fibrinolytic pathway secondary to increased amount of free thrombin which has not been neutralized by antithrombin [10].

In the current study, antiFXa levels were found to be higher in non-survivors as compared to survivors. Most studies that monitored heparin therapy by antiFXa assay in COVID-19 patients have reported suboptimal levels, suggesting heparin resistance [8,13,16-18]. It has also been suggested that since baseline hemostatic potential is markedly elevated in COVID-19 patients, heparin therapy is not as effective in decreasing the thrombin generating potential of plasma as it is in non-COVID-19 patients despite adequate levels [19]. Von Meijenfedt et al also reported low ATIII and increased antiFXa activity in non-survivors as compared to survivors. In their study, increased antiFXa levels correlated with decreased ex vivo thrombin generation [9]. Blasi et al reported in vitro normal thrombin generation despite detectable anti‐Xa activity in the majority of COVID-19 patients but enhanced in vivo thrombin generation as evidenced by elevated levels of thrombin‐antithrombin complexes and D‐dimers [20]. In the current study, increased antiXa activity can also be attributed to decreased heparin clearance due to dynamic fluctuations in the clinical profile of patients due to their inflammatory and hypercoagulable state. Considerable uncertainty still exists regarding the ideal assay for monitoring heparin therapy [6]. The inherent limitations of antiFXa assay must be kept in mind while interpreting the results. It is an indirect measurement of heparin levels in plasma. Those antiFXa assays in which antithrombin is added to the reagent mixture may overestimate antiFXa and may not be truly indicative of adequate in vivo anticoagulation, especially if ATIII titres are low [19]. The values of antiFXa assay may also be spuriously high or low if the timing of sample collection is not adhered to, which is sometimes difficult in clinical practice [21].

The limitations of the study include small sample size, retrospective study design and lack of serial measurements of fibrinogen, other coagulation factors and thrombin generation tests. Nevertheless, serial measurements of ATIII, antiFXa and other hemostatic parameters were compared between survivors and non-survivors, and reduced ATIII level in critically ill COVID-19 patients was found to be a poor prognostic indicator.

## Conclusions

To conclude, the results of our study suggest that depletion of ATIII may play a pivotal role in the progression of COVID-19-associated coagulopathy. These findings if substantiated by other studies may have important therapeutic and prognostic implications. The role of ATIII concentrate in selected cases needs to be explored after weighing the pros and cons of bleeding versus thrombosis. Since the kinetics of the coagulation cascade are complex, the results of coagulation assays need to be interpreted in the appropriate clinical context. Larger prospective studies incorporating serial measurements of global coagulation assays should be performed for further elucidating the pathophysiology of COVID-19- associated coagulopathy. Further, the utility of monitoring LMWH therapy by antiFXa assay needs to be determined.
